# Synergistic effect of *Euphorbia kansui* stir-fried with vinegar and bile acids on malignant ascites effusion through modulation of gut microbiota

**DOI:** 10.3389/fphar.2023.1249910

**Published:** 2023-11-09

**Authors:** Shengyun Dai, Shikang Zhou, Yonghui Ju, Weifeng Yao, Yuping Tang, Jian Zheng, Shuangcheng Ma, Yi Zhang, Li Zhang

**Affiliations:** ^1^ Institute for Control of Chinese Traditional Medicine and Ethnic Medicine, National Institute for Food and Drug Control, Beijing, China; ^2^ Jiangsu Key Laboratory for High Technology Research of TCM Formulae, National and Local Collaborative Engineering Center of Chinese Medicinal Resources Industrialization and Formulae Innovative Medicine and Jiangsu Collaborative Innovation Center of Chinese Medicinal Resources Industrialization, Nanjing University of Chinese Medicine, Nanjing, China; ^3^ School of Medicine and Chemical Materials, Zhenjiang College, Zhenjiang, China; ^4^ College of Pharmacy and Shaanxi Collaborative Innovation Center of Chinese Medicinal Resources Industrialization, Shaanxi University of Chinese Medicine, Xi’an, China

**Keywords:** *Euphorbia kansui*, malignant ascites, bile acids, gut microbiota, water-expelling effect

## Abstract

**Background:** Toxic *Euphorbia kansui* (EK) is employed to treat malignant ascites effusion (MAE). EK stir-fried with vinegar (VEK) has been demonstrated to reduce toxicity due to its preserved water-expelling effect. This was demonstrated to be correlated with gut microbiota. Therein, bile acids (BAs) have a bidirectional relationship with the gut microbiota. Therefore, the aim of this study is to explore whether BA-mediated gut microbiota influences the water-expelling effect of VEK against MAE.

**Methods:** The MAE rat model was established by intraperitoneal injection of Walker-256 tumor cells. A reliable simultaneous method for the determination of 15 bile acids in rat feces using ultra-high-performance liquid chromatography-tandem mass spectrometry (UPLC-MS/MS) was established and applied to analyze the fecal BAs in rats treated with VEK. The screened BA was then administered to VEK-treated MAE rats. The water-expelling effect was evaluated using histopathological analysis, biochemical examination, inflammatory factors in ascites, urine volume, ascites amount, and intestinal aquaporin expression. The microbial composition was determined using 16S rRNA sequencing, and the contents of bile acids were finally measured.

**Results:** VEK decreased the content of fecal deoxycholic acid (DCA), lithocholic acid (LCA), and taurocholic acid (TCA) while increasing the content of ursodeoxycholic acid (UDCA). VEK alleviated liver, stomach, and intestinal injuries; oxidative damage; and inflammation, which were further ameliorated with UDCA intervention. VEK alleviated MAE by increasing the fecal water content, urine volume, and AQP3 protein expression and decreasing the urine levels of Na^+^, K^+^, and Cl^−^. This was retained with the intervention of UDCA. UDCA and VEK regulated the BA metabolism disorder to a certain extent. Analysis of gut microbiota showed that VEK increased the abundance of *Lactobacillus* and decreased that of *Prevotella_9* in MAE rats. The combined administration of UDCA and VEK showed a better modulation of the microbiota structure than that of VEK alone, and the effect of this administration reached closer to the reference state.

**Conclusion:** The water-expelling effect of VEK did not directly depend on the BA-mediated gut microbiota. However, VEK and BAs had a synergistic effect on malignant ascites effusion through the regulation of the gut microbiota. These results provided a scientific basis for the reasonable usage of VEK and the novel combination treatment strategy of VEK and UDCA.

## 1 Introduction

Malignant ascites effusion (MAE) is defined as the severe presence of abnormal fluid accumulation in the peritoneal cavity, which is commonly caused by different types of cancer, cirrhosis, and heart failure (Zhang et al., 2018; Zennaro et al., 2020). The occurrence of MAE represented late-stage carcinoma with a poor prognosis and severely affected the patients’ quality of life (Jiang et al., 2016). At present, diuretics, abdominal puncture, infusion of albumin, and intraperitoneal chemotherapy are the main measures for treating MAE (Jiang et al., 2016; Guo et al., 2021). However, the existing therapies exert certain inherent toxicity or side effects (Pang et al., 2017). Hence, there is an urgent demand for a safer and more effective method for exploring the effective treatment of MAE.

*Euphorbia kansui* (EK), a characteristic type of Chinese herbal medicine (CHM), has a long history of application in patients with edema and ascites. The serious toxicity of EK, which can cause hepatic and gastrointestinal injury, skin irritation, and inflammation, has seriously restricted its clinical applications (Pang et al., 2017; Zhang, Q. et al., 2021). Therefore, EK stir-fried with vinegar (VEK) has been commonly employed to reduce its toxicity while retaining the water-expelling activity. Previous studies demonstrated that the mechanism of toxicity reduction while retaining efficacy is closely related to the gut microbiota and the interaction between the gut microbiota and the metabolism of the host, such as the change in the abundance of beneficial bacteria *Lactobacillus* and the contents of fecal short-chain fatty acids (SCFAs) (Jiang et al., 2018; Guo et al., 2021). However, the pharmacological mechanism of how VEK treated ascites through regulating gut microbiota and its co-metabolism with the host remains unclear.

Apart from SCFAs, bile acids (BAs) are another class of metabolites representing the interaction between the gut microbiota and the host metabolism. Bile acids can be classified into two types based on their origin (Behr et al., 2019): Ⅰ. the primary bile acids, such as cholic acid (CA), chenodeoxycholic acid (CDCA), α-muricholic acid (*α*-MCA), and *β*-muricholic acid (*β*-MCA), which are synthesized in the liver and transported to the intestine to form the secondary bile acids after the modification of the gut microbiota, which in turn dominate in the total fecal bile acid pool. Ⅱ. The secondary bile acids, such as deoxycholic acid (DCA), lithocholic acid (LCA), ursodeoxycholic acid (UDCA), and *ω*-muricholic acid (*ω*-MCA). BAs and gut microbiota are interdependent and competitive. BAs act as important endogenous signaling molecules that may regulate glucose, lipid, and energy metabolism. They play an important role in the occurrence of liver disease, inflammatory bowel disease, metabolic syndrome, cancer, and other diseases (Vítek and Haluzík, 2016; Vaz and Ferdinandusse, 2017). The gut microbiome can affect bile acid distribution by regulating the expression of the farnesoid X receptor (FXR), which is activated by bile acids via a negative feedback mechanism (Staley et al., 2017). Conversely, some bile acids have a bacteriostatic action, and changes in the bile acid profile affect the gut microbiota population (Behr et al., 2019). When large amounts of ascites are produced, the balance between the beneficial and harmful gut microbiota in the intestine is disrupted. The gut barrier is further damaged, and the inherent immune system is activated, accompanied by the release of inflammatory factors. Meanwhile, the abnormal metabolism of BAs and the inhibition of FXR increase intestinal permeability, leading to bacterial translocation and abnormal excretion of albumin. The production of ascites was finally agrregated (Schwabl et al., 2017; Muñoz et al., 2019).

In recent years, BAs have been well determined by ultra-high-performance liquid chromatography-tandem mass spectrometry (UPLC-MS/MS). UPLC-MS/MS could realize the detection of different BAs with great sensitivity and easy sample pretreatment (Franco et al., 2019). Therefore, in this study, UPLC-MS/MS was used to establish a quantitative method for the analysis of bile acids in rat feces to explore the changes in bile acid metabolism caused by the administration of VEK. Then, based on bile acids intervening in MAE rats, the association between the water-expelling effect of VEK and BA-mediated gut microbiota was further verified.

## 2 Materials and methods

### 2.1 Chemicals and reagents

Cholic acid (CA), chenodeoxycholic acid (CDCA), deoxycholic acid (DCA), lithocholic acid (LCA), hyodeoxycholic acid (HDCA), ursodeoxycholic acid (UDCA), glycocholic acid (GCA), glycodeoxycholic acid (GDCA), taurocholic acid (TCA), tauroursodeoxycholic acid (TUDCA), *α*-muricholic acid (*α*-MCA), *β*-muricholic acid (*β*-MCA), and *ω*-muricholic acid (*ω*-MCA) were purchased from Yuanye Bio-Technology Co. (Shanghai, China). Tauro-α-muricholic acid (T-*α*-MCA) and tauro-*β*-muricholic acid (T-*β*-MCA) were purchased from Zhenzhun Biotechnology Co. (Shanghai, China). Glycocholic-2,2,4,4-d4 acid (GCA-d4) was obtained from IsoSciences (Ambler, United States). The purity of the aforementioned standard substances was above 98%. Methanol, acetonitrile, and formic acid of HPLC grade were purchased from Merck Co. (Darmstadt, Germany). All other reagents were of analytical grade.

### 2.2 Plant materials

The roots of *Euphoria kansui* were collected from Baoji, Shaanxi Province, China. The crude drug was identified by Prof. Qinan Wu (Nanjing University of Chinese Medicine, Nanjing, 210023, China), and a voucher specimen (No. NJUTCM-20171015) was deposited in the Herbarium of Nanjing University of Chinese Medicine (Nanjing, China). According to the previous processing method, 100 g of EK was immersed in 30 g of vinegar until fully absorbed. Then, it was stir-fried for approximately 9 min at 260°C until slightly scorched spots appeared (Zhang et al., 2019). The dried EK and VEK were ground into a powder and dispersed in a 0.5% CMC-Na solution with a concentration of 68 g·L^−1^, respectively. The quality of EK and VEK was evaluated using the methods pre-established in the laboratory (Zhang et al., 2018).

### 2.3 Animals and treatment

**FIGURE 1:**
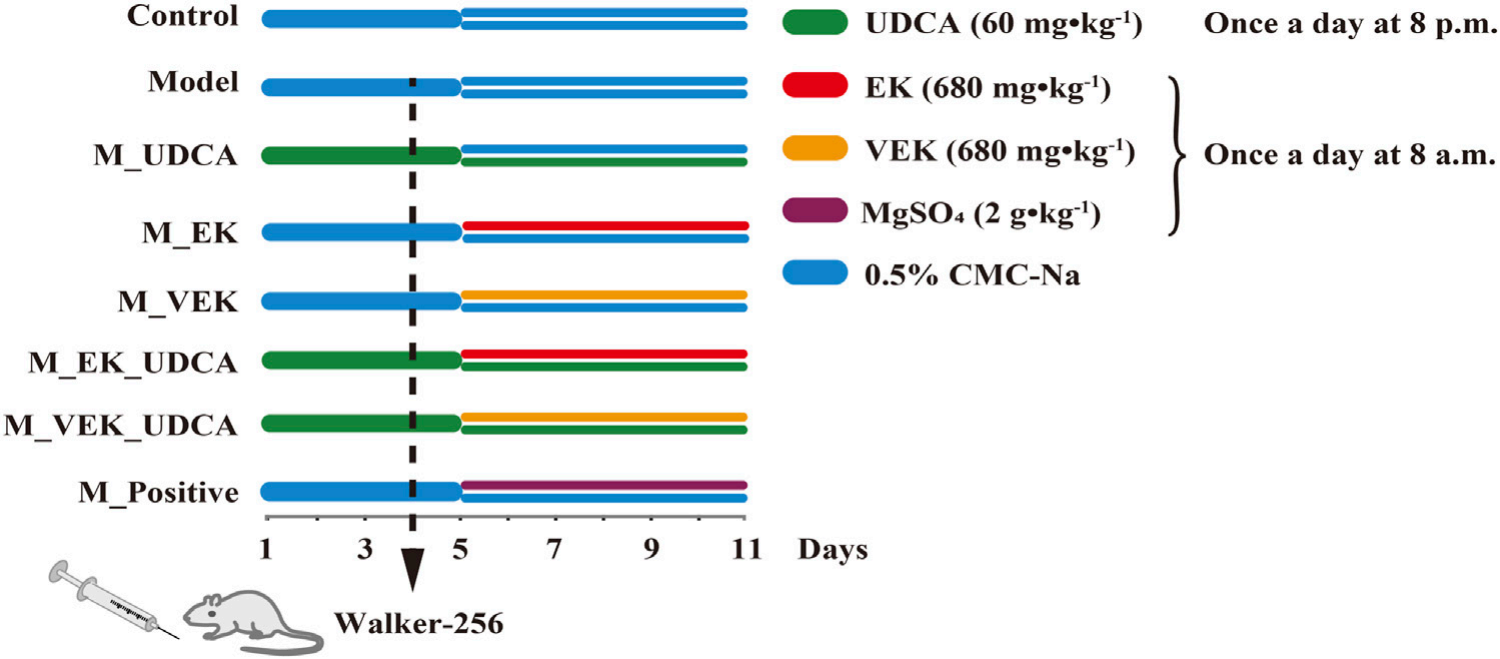
Rat dosing regimen.

A total of 24 Sprague–Dawley (SD) male rats (230 ± 20 g) were housed at a certified animal experimental laboratory with a 12-h light/dark cycle and a constant temperature of 25°C ± 1°C. The animals were allowed free access to food and water. After acclimatization for 1 week, the rats were randomly divided into four groups: control group, model group, MEK group, and MVEK group. The normal rats in the control group were administered 0.5% CMC-Na by oral gavage. The rats in the other groups were all intraperitoneally injected with Walker-256 tumor cells to induce MAE rats (Shen et al., 2016). MAE rats were then treated with 0.5% CMC-Na (model group), the EK powder at a dosage of 680 mg·kg^−1^ (MEK group), and the VEK powder at a dosage of 680 mg·kg^−1^ (MVEK group) for 7 consecutive days. Fecal samples were collected on day 6 after administration, then frozen immediately in liquid nitrogen and stored at −80°C for bile acid analysis (Guo et al., 2021). Furthermore, BAs were selected, and a synergistic intervention with EK/VEK was carried out to verify the influence of BA-mediated gut microbiota on the water-expelling effect of EK/VEK. A total of 64 SD male rats were randomly divided into eight groups: control group, model group, EK group (M_EK), VEK group (M_VEK), UDCA group (M_UDCA), EK + UDCA group (M_EK_UDCA), VEK + UDCA group (M_VEK_UDCA), and positive group (MgSO_4_ at a dosage of 2 g·kg^−1^). The administration mode is shown in Figure 1. Animal care was performed according to the Guidelines for Animal Experimentation of Nanjing University of Chinese Medicine. The protocols were approved by the Animal Ethics Committee of the University (202004A019).

### 2.4 Measurement of the water content of feces; urine volume; Na^+^, K^+^, and Cl^−^ levels; and the amount of ascites

On day 10, the rats were placed in metabolic cages, and fresh feces samples were collected for 6 h. The moisture content of the feces was calculated as follows: (wet weight of feces−dry weight of feces)/wet weight of feces × 100%. In addition, the urine samples were collected for 24 h. The urine volume was measured, and Na^+^, K^+^, and Cl^−^ levels were determined according to the specifications of the corresponding commercial kits. The amount of ascites was calculated as the difference between the two weights after the ascites were taken.

### 2.5 Biochemical examinations

The detection kits for blood biochemistry, which include alanine aminotransferase (ALT), aspartate aminotransferase (AST), glutathione (GSH), malondialdehyde (MDA), and superoxide dismutase (SOD), were all purchased from Jiancheng Bioengineering Institute (Nanjing, China). On day 11, all rats were anesthetized with 6% sodium pentobarbital (90 mg·kg^-1^), and plasma was collected via the carotid artery for serum collection. The levels of ALT and AST in the serum were determined using an automatic biochemical analyzer (AU680; Beckman Coulter, Inc., United States). In addition, the liver and jejunum tissues were collected, and the appropriate amount of tissue was homogenized. The supernatant was then collected after centrifugation for the determination of SOD, GSH, and MDA.

### 2.6 Histological observation

An appropriate amount of liver, stomach, and jejunum tissues was removed, fixed in paraformaldehyde (4%), and embedded in paraffin. Hematoxylin and eosin (HE) staining of the paraffin-embedded sections was performed. The slices were examined using a microscope to observe pathological differences.

### 2.7 Enzyme-linked immunosorbent assay

The levels of TNF-α and IL-6 in ascites were quantified using ELISA kits (Jiancheng, Nanjing, China). All operations in the experimental process were carried out according to the manufacturer’s specifications.

### 2.8 Immunohistochemistry

The intestinal sections were deparaffinized in xylene and washed in a gradient concentration (pH = 6.0) of ethanol. Then, the antigen composed of tissues was repaired using a steamer in citrate buffer for 20 min. The sections were incubated in H_2_O_2_ (3%) at room temperature for 25 min in the dark to block endogenous peroxidase using bovine serum albumin (BSA) for 30 min at 37°C. Then, they were incubated in H_2_O_2_ (3%) at room temperature for 10 min to block endogenous peroxidase. Thereupon, the slides were incubated at 4°C with the primary antibody (AQP3 1:200 dilution) for 2 h. Next, the slides were washed with phosphate-buffered saline (PBS) three times and incubated with HRP-conjugated goat anti-rabbit IgG (1:200 dilution) for 30 min at 37°C. After further washing with PBS, the slides were stained with diaminobenzidine (DAB) to visualize the target signals and counterstained with hematoxylin to visualize the cell nuclei. The mean density of the target signals was analyzed using Image-Pro Plus 6.0 (IPP 6.0, Media Cybernetics, Silver Spring, MD, United States) software on the acquired images.

### 2.9 Quantification of bile acids in rat feces

#### 2.9.1 Preparation of sample solutions

A measure of 50 mg of the fecal sample was accurately weighed and added to 200 µL of ice water and 10 µL of the internal standard (IS). The mixture was vortexed for 30 s, followed by homogenization for 3 min; 100 μL of the supernatant was obtained by centrifugation at 13,000 *g* for 20 min. The precipitate was extracted with 200 μL of ice methanol by homogenization for 3 min, and 100 µL of the supernatant was obtained. The supernatants obtained from the first and second extractions were mixed, vortexed, and centrifuged at 13,000 g for 10 min; 100 μL of the supernatant was transferred into another centrifuge tube and evaporated to dryness. The residue was re-dissolved with 800 μL of methanol/water (50:50, *v*:*v*), centrifuged at 13,000 g for 10 min, and the supernatant was finally used for analysis.

#### 2.9.2 Preparation of standards, calibration standards, and quality control samples

Standard stock solutions containing *α*-MCA (1), *β*-MCA (2), *ω*-MCA (3), UDCA (4), HDCA (5), CA (6), LCA (7), DCA (8), CDCA (9), T-*α*-MCA (10), T-*β*-MCA (11), TCA (12), GCA (13), GDCA (14), and TUDCA (15) were prepared by dissolving the accurately weighed corresponding standard references in methanol, followed by dilution to final concentrations of 1.080 mg·mL^−1^, 1.000 mg·mL^−1^, 1.000 mg·mL^−1^, 0.9500 mg·mL^−1^, 1.340 mg·mL^−1^, 1.460 mg·mL^−1^, 0.9800 mg·mL^−1^, 1.110 mg·mL^−1^, 0.9900 mg·mL^−1^, 1.000 mg·mL^−1^, 1.520 mg·mL^−1^, 1.020 mg·mL^−1^, 1.050 mg·mL^−1^, 1.520 mg·mL^−1^, and 1.020 mg·mL^−1^, respectively. GCA-d4 was dissolved in methanol and further diluted to 3.180 μg/mL to obtain the IS solution.

Seven aliquots of the blank fecal sample were spiked with a mixed standard solution of 15 BAs to prepare the calibration standards with the concentrations given in Tables 2, 3. Quality control (QC) samples that included low, medium, and high concentrations for each analyte were independently prepared similarly.

#### 2.9.3 Instrumentation and conditions of UPLC-MS/MS

The Shimadzu LC-20 series (Shimadzu, Japan) and a QTRAP 5500 mass spectrometer (AB Sciex, United States) coupled with an electrospray ionization (ESI) source were used for quantitative analysis. The Waters XBridge BEH Shield RP18 (Milford, United States) column (100 mm × 2.1 mm; 2.5 μm) was employed for chromatographic separation, and the column temperature was 40°C. The mobile phase consisted of 0.2% formic acid in the aqueous solution (A) and 0.2% formic acid in acetonitrile (B) with the following gradient elution: 0–2 min, 20%–20% B; 2–4 min, 20%–53.5% B; 4–9 min, 53.5%–54.5% B; 9–11 min, 54.5%–56% B; 11–13 min, 56%–85% B; 13–14 min, 85%–90% B; 14–16 min, 90%–20% B; and 16–18 min, 20%–20% B. The liquid flow rate was 0.45 mL·min^−1^, and the injection volume was 2 μL.

The negative mode was employed for detecting BAs with a mass range of 50 to 1,500. The ion source temperature and spray voltage were 550°C and −4,500 V, respectively. The pressures for the curtain gas and heater gas were 227.568 KPa and 379.28 KPa, respectively. The ion source gas for nebulization was set at 379.28 KPa. The MRM parameters of each analyte were optimized, as summarized in Table 1.

**TABLE 1:** Optimum MRM conditions.

	Parent (m/z)	Daughter (m/z)	DP/V	CE/V	CXP/V
α-MCA	407.263	407.263	−75	−20	−9
β-MCA	407.263	407.263	−75	−20	−9
ω-MCA	407.263	407.263	−75	−20	−9
UDCA	391.228	373.1	−230	−42	−25
HDCA	391.261	373.1	−230	−42	−25
CA	407.199	343	−250	−44	−37
LCA	375.225	375.225	−150	−10	−29
DCA	391.261	373.1	−230	−42	−25
CDCA	391.219	373.1	−230	−42	−25
T-β-MCA	514.142	79.8	−130	−130	−11
T-α-MCA	514.142	79.8	−130	−130	−11
TCA	514.142	79.8	−130	−130	−11
GCA	464.188	74	−200	−92	−9
GDCA	448.197	74.1	−100	−68	−9
TUDCA	498.133	80	−35	−128	−9
GCA-d4	468.208	74	−110	−96	−9

#### 2.9.4 Method validation

##### 2.9.4.1 Linear range, limits of detection, and limits of quantification

The linearity was evaluated at seven concentrations of mixed standard solutions. Y represents the peak area (well resolution)/peak height (poor resolution) ratios of each analyte to IS, and X represents the concentrations of the analyte (X). The equation was further established using the weighted least squares (1/X^2^) linear regression analysis. For each constituent, the limits of detection (LOD) and limits of quantification (LOQ) were determined by the serial dilution of the standard solution. LOD and LOQ were calculated based on the peak-to-noise ratios of 3:1 and 10:1, respectively.

##### 2.9.4.2 Accuracy and precision

The precision of the method was evaluated by intra-day and inter-day variations. Intra-day precision and accuracy were assessed by five replications of the high, medium, and low levels of the quality control samples within one day. The inter-day precision was assessed by three replications of the high, medium, and low levels of the quality control samples for three consecutive days.

##### 2.9.4.3 Stability

The short-term stability was evaluated by keeping the QC samples at an ambient temperature for 24 h. The long-term stability was assessed by placing the samples at −80°C for 15 days. During freeze–thaw stability evaluations, the samples were analyzed after undergoing three freeze–thaw cycles (from −80°C to room temperature). The stability of analytes was evaluated using the RSD values of replicated samples.

##### 2.9.4.4 Extraction recovery and matrix effect

The extraction recovery of the method was evaluated using five replicates at three concentration levels of high, medium, and low levels by comparing the peak areas of the 15 analytes in the fecal blank spiked samples to the peak areas of the extracted spiked samples. The matrix effect was measured at three concentration levels by comparing the peak areas of the analytes and the IS solution from the post-extraction spiked blank samples to those of the methanol aliquots spiked with the pure reference standard.

### 2.10 Analysis of fecal 16S rRNA

Fresh fecal samples were collected on day 10 for the gut microbial analysis. The sequencing of fecal samples was supported by Majorbio Biotechnology Co., Ltd., Shanghai, China. Bacterial genomic DNA was extracted from frozen fecal samples stored at −80°C using the MN NucleoSpin 96 Soil Kit (Clontech, Takara, Japan). The 16S rRNA gene comprising the V3 and V4 regions was amplified by PCR using the following common primers: primer 338F: 50-ACTCCTACGGGRSGCAGCAG-30; primer 806R: 50-GGACTACVVGGGTATCTAATC-30. The amplicon quality was evaluated using gel electrophoresis. The PCR products were purified using the AxyPrep DNA Gel Extraction Kit (Axygen Biosciences, United States) and quantified using the QuantiFluor™-ST system. Sequencing was performed on an Illumina MiSeq platform at Biomarker Technologies Co., Ltd. (Beijing, China) with two 300-base paired-end read cycles of each.

### 2.11 Statistical analysis

The numerical data were expressed as means ± standard deviation. All statistical analyses were performed using GraphPad Prism 7. Comparisons among experimental groups were carried out using a one-way analysis of variance (ANOVA), followed by the least significant difference *t*-test. *p*-values <0.05 were considered statistically significant, and *p*-values <0.01 were considered highly statistically significant.

## 3 Results

### 3.1 Optimization of UPLC-MS/MS conditions

**FIGURE 2:**
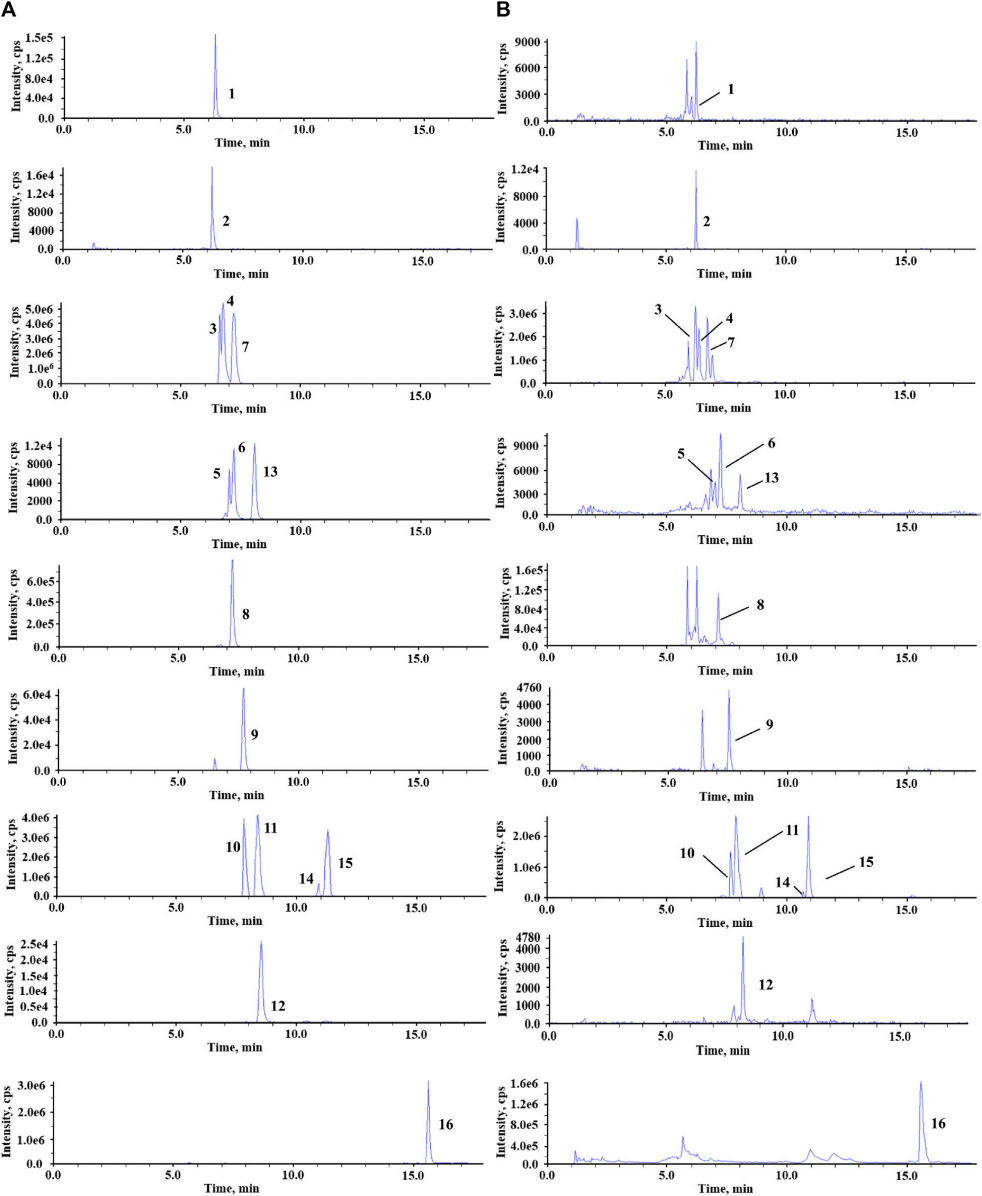
Representative MRM chromatograms of standard solutions **(A)** and fecal sample **(B)**. 1, GCA; 2, GCA-d4; 3, ω-MCA; 4, α-MCA; 5, T-α-MCA; 6, T-β-MCA; 7, β-MCA; 8, CA; 9, GDCA; 10, UDCA; 11, HDCA; 12, TUDCA; 13, TCA; 14, DCA; 15, CDCA; 16, LCA.

To achieve better performance in the separation of different BAs, especially for the conformational isomers with the same ion pair, the chromatographic conditions were optimized. The water–acetonitrile system showed a better separation resolution for bile acids. Because two epimoric pairs T-*α*/*β*-MCA and *α*/*ω*-MCA were not fully resolved, peak height ratios (target bile acid to IS) were taken for linear regression. Other bile acids were fully resolved, and the corresponding linear regressions were performed using the peak area ratio (target bile acid to IS). At the same time, the addition of formic acid improved the peak shape and enhanced the signals of BAs. The positive and negative ion modes were then carried out for mass spectrometry. A total of 15 BAs obtained a high-intensity and stable response in negative ion modes. Therefore, the negative ion mode was selected. The [M–H]^-^ ions were chosen as precursor ions for detecting analytes, and mass spectrum parameters were optimized for the most intense product ion including collision energy (CE), collision exit potential (CXP), and declustering potential (DP) (Table 1). The MRM chromatograms for the standard solutions of each analyte are shown in Figure 2.

### 3.2 Method validation

A total of 15 compounds showed good linearity within the range of 0.49 to 20,100 ng·mL^−1^. The LOD and LOQ were in the range of 0.01–0.51 ng·mL^−1^ and 0.04–1.9 ng·mL^−1^, respectively (Tables 2, 3). The RSD values of intra-day and inter-day precisions ranged from 2.7% to 14.5%, which indicated that the precision of this method was acceptable (Supplementary Table S1). In addition, these compounds were stable under four storage conditions (Supplementary Table S2). The extraction recoveries of these compounds ranged from 80.7% to 114.6%, with RSD values lower than 15%, demonstrating that the method was accurate and feasible (Supplementary Table S3).

**TABLE 2:** Investigation of the linear correlation (Y, peak area ratios of the analytes to IS).

Compound	Calibration equation	Correlation coefficient (r)	Linear range (ng/mL)	LOQ (ng/mL)	LOD (ng/mL)
β-MCA	y = 0.483x + 32.762	0.995	39.06–2,500	0.35	0.10
UDCA	y = 0.188x + 5.619	0.996	55.66–3,562	1.50	0.45
HDCA	y = 0.034x + 94.461	0.996	314.06–20,100	0.87	0.30
CA	y = 0.035x + 0.473	0.994	45.63–2,920	0.58	0.17
LCA	y = 0.112x + 3.506	0.992	76.56–4,900	1.9	0.51
DCA	y = 0.044x + 1.968	0.994	173.44–11,100	0.79	0.28
CDCA	y = 0.168x + 0.093	0.996	3.87–247.5	0.27	0.15
TCA	y = 0.034x + 0.018	0.994	1.00–63.75	0.06	0.01
GCA	y = 0.118x − 0.03	0.998	1.64–105	0.19	0.06
GDCA	y = 0.118x − 0.009	0.998	1.19–76	0.12	0.04
TUDCA	y = 0.076x + 8.903E^-5^	0.991	1.00–63.75	0.07	0.03

**TABLE 3:** Investigation of the linear correlation (Y, peak height ratios of the analytes to IS).

Compound	Calibration equation	Correlation coefficient (r)	Linear range (ng/mL)	LOQ (ng/mL)	LOD (ng/mL)
α-MCA	y = 0.082x + 22.464	0.990	126.56–8,100	0.83	0.25
ω-MCA	y = 0.207x + 8.367	0.991	39.06–2,500	0.37	0.11
T-α-MCA	y = 0.029x + 0.023	0.990	0.49–31.25	0.04	0.01
T-β-MCA	y = 0.018x + 0.035	0.991	1.48–95	0.09	0.02

### 3.3 Determination of fecal bile acids in EK/VEK-treated MAE rats

**FIGURE 3:**
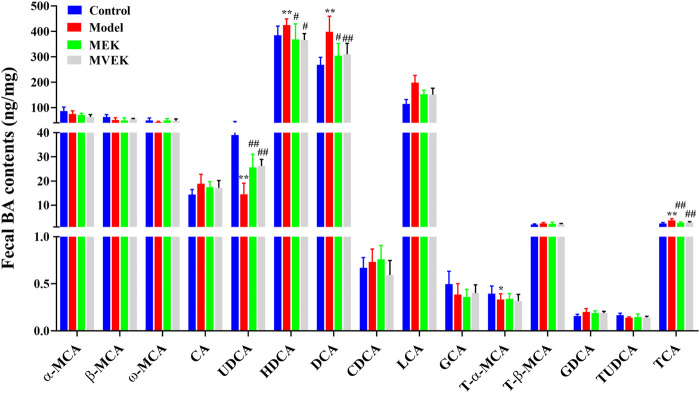
Effects of EK and VEK on the BA content in the feces of model rats(*x̄* ± s, *n* = 6)

To investigate whether the fecal bile acid changes affect the water-expelling effect of EK and VEK, the contents of 15 BAs from normal rats, MAE rats, and rats administered with EK/VEK were analyzed. In Figure 3, compared with the control group, DCA, LCA, and TCA in the model group increased significantly (*p* <0.01), while UDCA decreased significantly (*p* <0.01). No significant change was observed for other bile acids. Compared with the model group, DCA, LCA, and TCA all decreased to varying degrees, and UDCA increased significantly (*p* <0.05) in the MEK and MVEK groups. Hence, in the subsequent validation experiment, UDCA was selected to further investigate whether BA-mediated gut microbiota influenced the treatment of EK/VEK.

### 3.4 Evaluation of HE staining in EK/VEK-treated MAE rats with UDCA intervention

**FIGURE 4:**
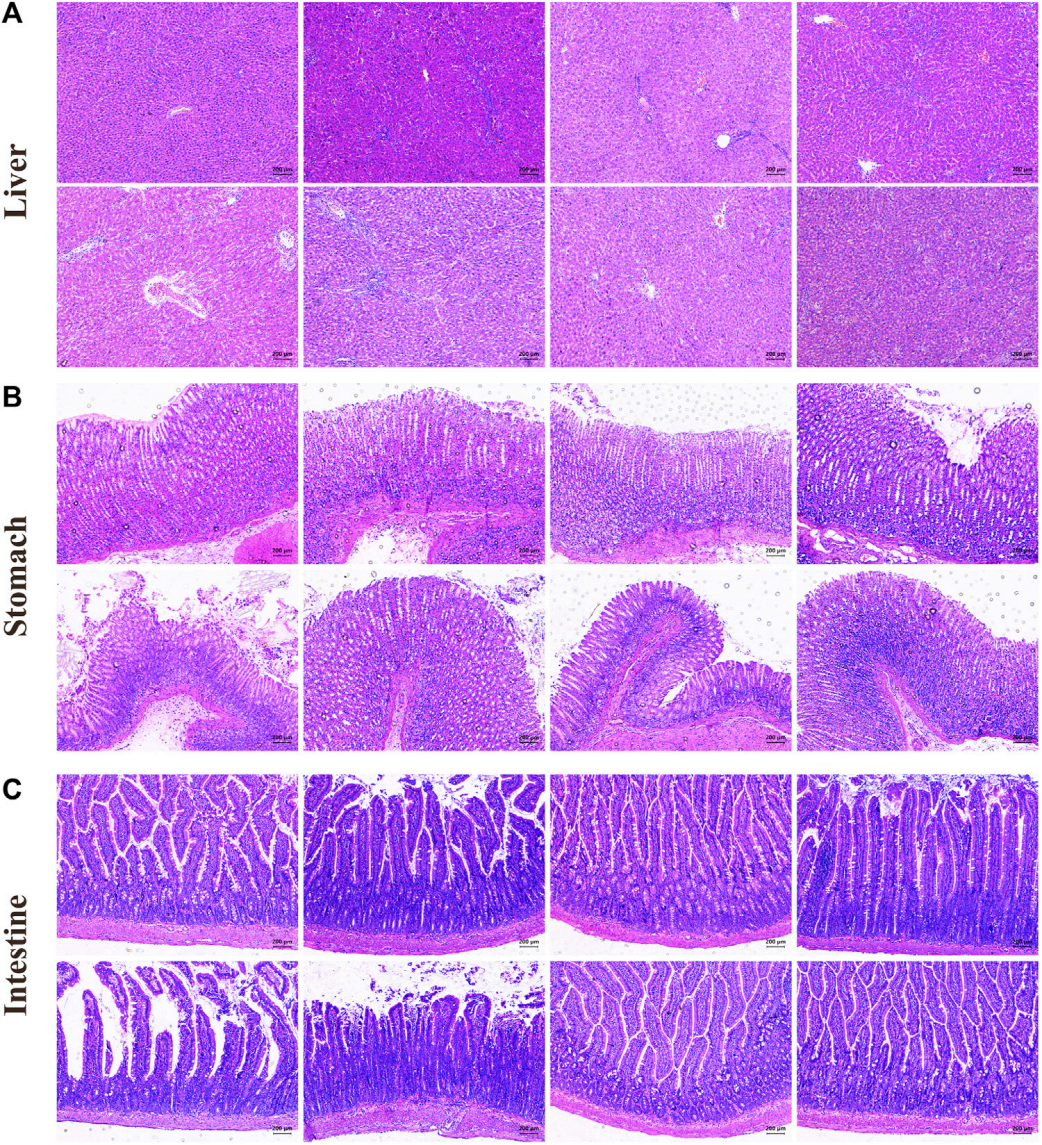
HE staining of the liver **(A)**, stomach **(B)**, and intestine **(C)** (magnification, ×100; control: rats with no treatments, model: model rats with Walker-256 malignant ascites effusion, M_UDCA: model rats treated with UDCA, M_EK: model rats treated with EK, M_VEK: model rats treated with VEK, M_EK_UDCA: model rats treated with EK and UDCA, M_VEK_UDCA: model rats treated with VEK and UDCA, and positive: model rats treated with MgSO_4_).

In Figure 4A, the liver structure of the control group rats was clear and normal. Compared with the control group, the hepatocytes of the model group showed enlarged spaces, an irregular arrangement, and pathological changes such as inflammatory cell infiltration and dissolution. EK and VEK could alleviate the aforementioned injuries in MAE rats. Compared with the M_EK and M_VEK groups, respectively, damage to hepatocytes in the M_EK_SCFA and M_VEK_SCFA groups was further relieved. In Figure 4B, the gastric mucosal epithelial structure appeared to be intact, and the glands in the mucosa were closely arranged for rats in the control group. Compared to the control group, the gastric epithelial cells suffered severe erosion and shedding accompanied by inflammatory infiltration in the model group. Compared to the model group, the gastric damage in the M_UDCA group was slightly alleviated. EK and VEK relieved the gastric epithelial damage. In Figure 4C, the intestinal mucosa structure of the control group was complete, and the intestinal villi cells looked to be arranged normally. Compared with the control group, the intestinal villi cells in MAE rats were severely ruptured with obvious inflammatory infiltration. Compared with the model group, EK and VEK significantly improved the intestinal injury, while UDCA slightly improved. Compared with the M_EK and M_VEK groups, respectively, the intestinal inflammation was better relieved after the intervention of UDCA. The aforementioned results indicate that there were obvious liver, stomach, and intestinal injuries in the model group, and these organic injuries could be alleviated after the administration of EK and VEK. There was no significant difference between the effectivity of EK and VEK. When supplemented with UDCA, the protective effects of EK and VEK on the liver, stomach, and intestine in MAE rats were further strengthened.

### 3.5 Determination of liver function and oxidative damage in EK/VEK-treated MAE rats with UDCA intervention

**FIGURE 5:**
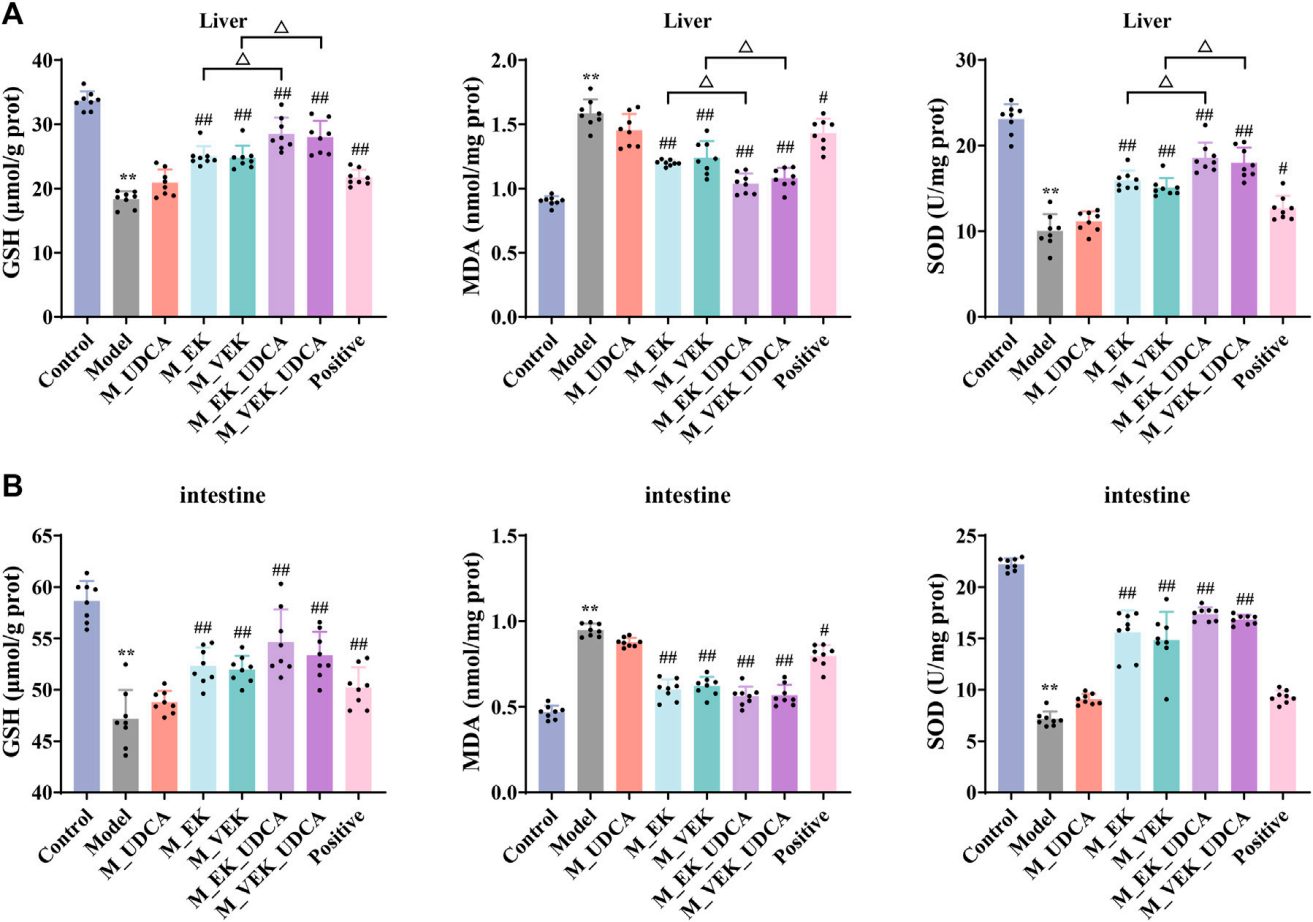
GSH, SOD, and MDA values in the liver **(A)** and intestine **(B)** of rats for each group (*x̄* ±s; *n* = 8); **p <0.01, compared to the control group; ^**#**^ *p* <0.05, ^**##**^ *p* <0.01, compared to the model group; ^Δ^ *P* <0.05, compared to EK; and ^▽^ *p* <0.05, compared to the VEK group.

In Table 4, compared to the control group, the levels of serum ALT and AST were significantly increased in MAE rats. Compared with the model group, the levels of serum ALT and AST were significantly reduced after treatment with EK/VEK. Compared with the M_EK and M_VEK groups, respectively, the intervention of UDCA further decreased the levels of serum ALT and AST. In addition, GSH, SOD, and MDA of the liver and intestinal homogenates were measured to evaluate the oxidative damage (Figure 5). MAE rats showed obviously decreased GSH and SOD levels and a significantly increased MDA level compared to the control group. Compared to the model group, GSH and SOD increased, while MDA decreased in the liver and intestinal homogenates when treated with EK and VEK. Compared to the M_EK and M_VEK groups, respectively, the levels of GSH, SOD, and MDA in the liver and intestinal homogenates further improved in the M_EK_UDCA and M_VEK_UDCA groups. The aforementioned results showed that EK and VEK obviously alleviated liver and intestine injuries in MAE rats, and this effectivity was further strengthened by UDCA intervention.

**TABLE 4:** Effects of different groups on the serum AST and ALT levels in rats (*x̄* ± s, *n* = 8).

Group	ALT (U•L^−1^)	AST (U•L^−1^)
Control	15.20 ± 2.73	18.60 ± 3.56
Model	35.31 ± 2.46**	42.60 ± 4.68**
M_UDCA	32.93 ± 1.77	38.54 ± 3.78
M_EK	24.31 ± 1.58^##^	30.72 ± 3.28^##^
M_VEK	25.22 ± 1.61^##^	32.81 ± 3.75^##^
M_EK_UDCA	20.72 ± 1.73^##Δ^	24.56 ± 2.64^##Δ^
M_VEK_UDCA	21.47 ± 1.60^##▽^	26.62 ± 2.93^##▽^
Positive	29.52 ± 2.67^#^	29.52 ± 3.58^##^

**p <0.01, compared to the control group; ^#^ *p* <0.05, ^##^ *p* <0.01, compared to the model group; ^Δ^ *p* <0.05, compared to EK; ^▽^ *p* <0.05, compared to the VEK group.

### 3.6 Determination of ascites volume, urine volume, fecal water content, and intestinal AQP3 expression in EK/VEK-treated MAE rats with UDCA intervention

**FIGURE 6:**
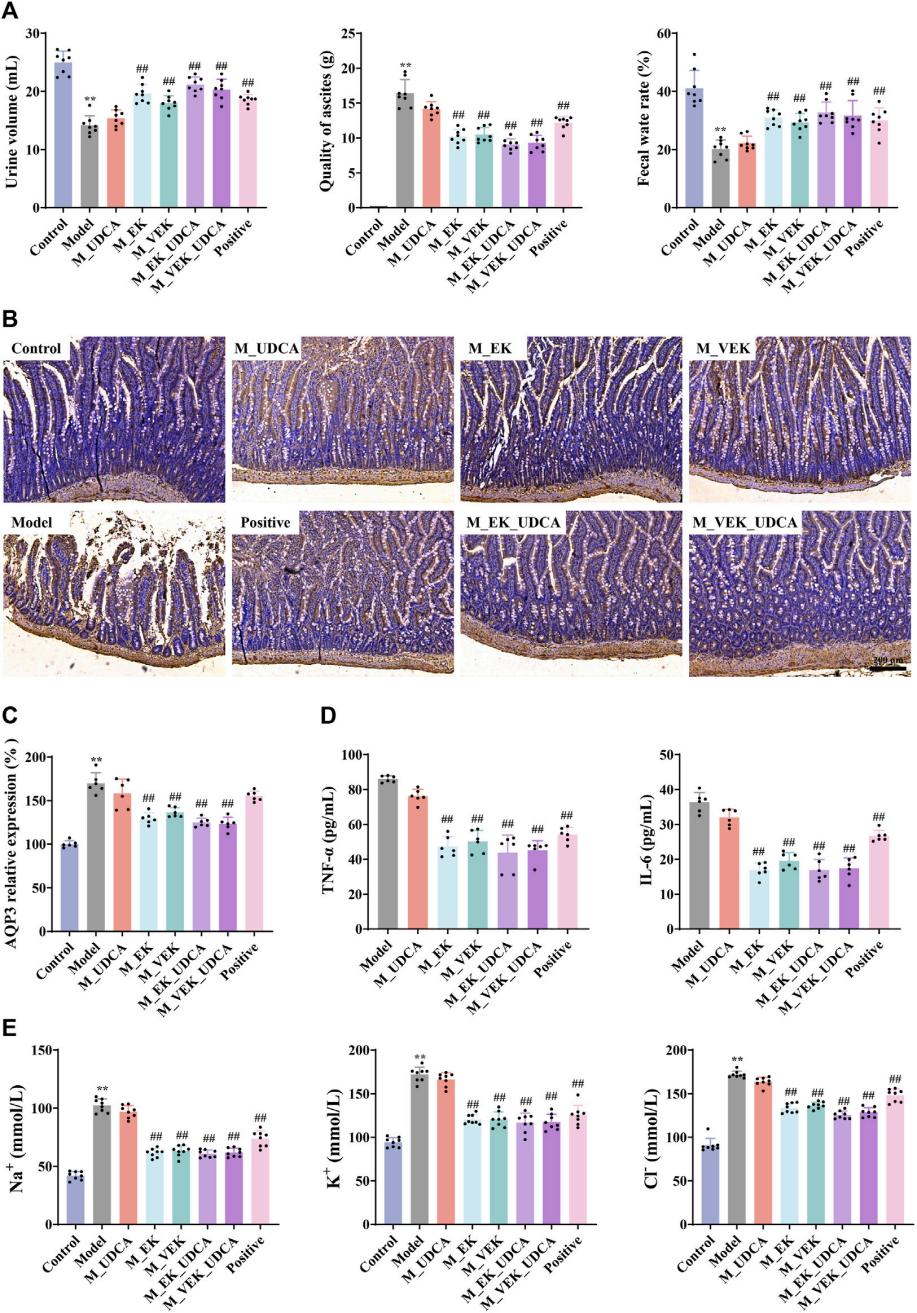
**(A)** Effects of each group on urine, ascites, and fecal water rate in rats (*x̄* ±s, *n* = 8); **(B, C)** IHC determined the AQP3 expression in the rat intestine (magnification, ×100; *x̄* ± s, *n* = 6); **(D)** effects of each group on TNF-α and IL-6 (*x̄* ±s, *n* = 6); **(E)** effects of different administration groups on urine Na^+^, K^+^, and Cl^−^ levels in rats (*x̄* ±s; *n* = 8). *p <0.05, ** *p* <0.01, compared to the control group; ^**#**^ *p* <0.05, ^**##**^ *p* <0.01, compared to the model group.

Compared to the normal rats, the MAE rats showed obvious ascites (Figure 6A), while their urine volume and fecal water rate were significantly decreased. Compared to the model group, the ascites volume was significantly reduced with an obvious increase in the urine volume and fecal water content when treated with EK/VEK. There was no significant difference between the EK and VEK groups. Compared with the M_EK and M_VEK groups, respectively, the aforementioned indicators in the M_EK_UDCA and M_VEK_UDCA groups improved slightly. In addition, the AQP3 protein was highly expressed in the model group compared with the control group. This trend was reversed by EK and VEK with the intervention of UDCA (Figures 6B, C). These results showed that both EK and VEK well exhibited the water-expelling effect by decreasing the expression of intestinal AQP3, and UDCA intervention could synergize with EK/VEK to exert effectivity against MAE.

### 3.7 Determination of urinary Na^+^, K^+^, and Cl^−^ in EK/VEK-treated MAE rats with UDCA intervention

Compared to the control group, the contents of urinary Na^+^, K^+^, and Cl^−^ were significantly increased in the model group (Figure 6E). After administration with EK and VEK, all these indicators were obviously reduced, and there was no significant difference between the EK and VEK groups. The results indicate that EK and VEK significantly alleviated the urinary electrolyte disturbances in MAE rats, and UDCA intervention had no interference with them. Compared with the M_EK and M_VEK groups, respectively, the aforementioned indicators in the M_EK_UDCA and M_VEK_UDCA groups showed no significant differences.

### 3.8 Determination of TNF-*α* and IL-6 in ascites of EK/VEK-treated MAE rats with UDCA intervention

In Figure 6D, compared to the model group, the contents of TNF-α and IL-6 in ascites were dramatically reduced in the EK/VEK-treated groups, and there was no significant difference between these groups. Compared with the M_EK and M_VEK groups, a decreasing trend of TNF-*α* and IL-6 was observed in M_EK_UDCA and M_VEK_UDCA groups, respectively. These results indicate that EK and VEK could relieve inflammation in ascites with the intervention of UDCA.

### 3.9 Determination of fecal bile acids in EK/VEK-treated MAE rats with UDCA intervention

**FIGURE 7:**
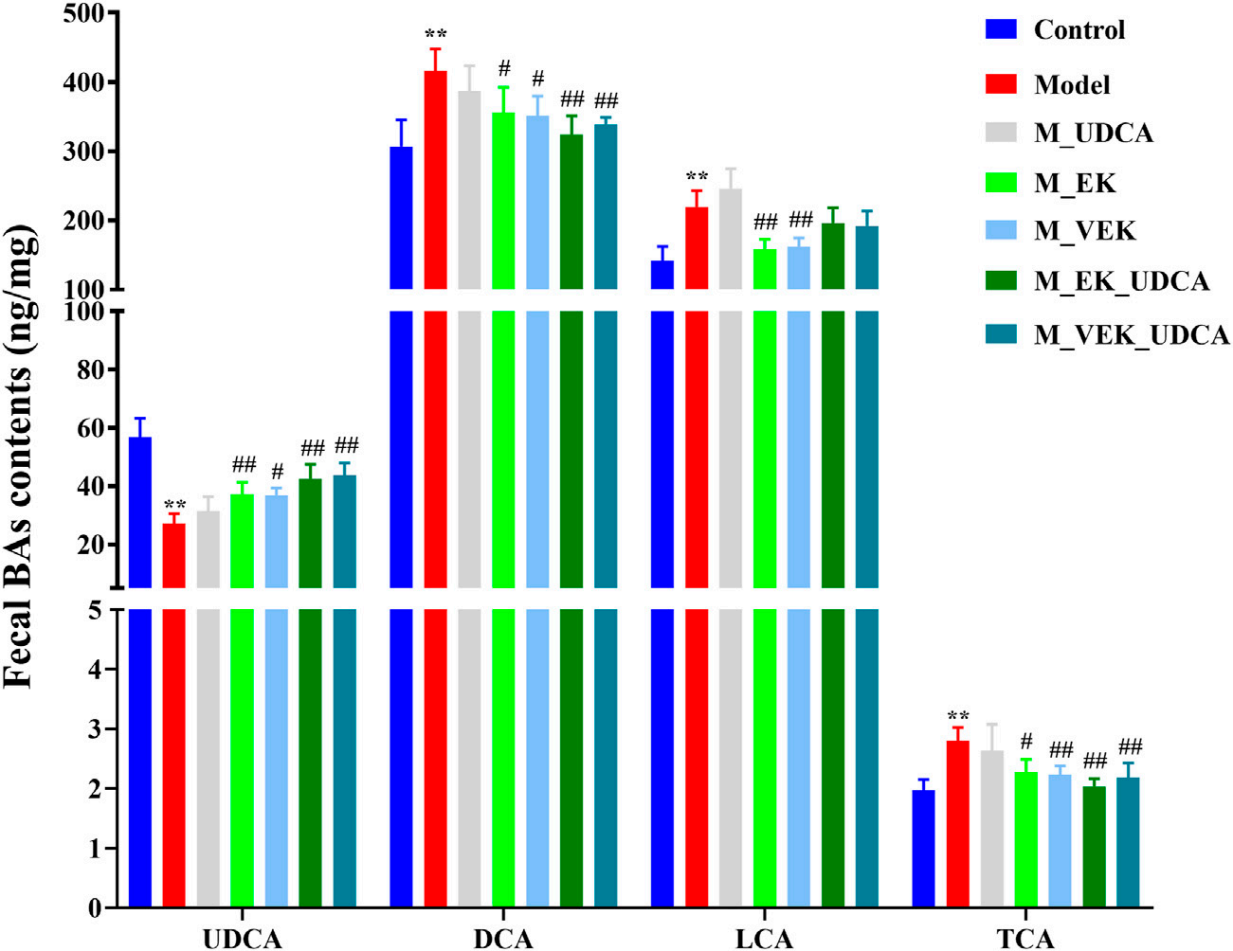
Effects of EK and VEK on the fecal bile acid profile in different groups (*x̄* ± s, *n* = 6); **p <0.01, compared to the control group; ^**#**^ *p* <0.05, ^**##**^ *p* <0.01, compared to the model group.

In Figure 7, compared with the control group, UDCA was significantly reduced and the levels of DCA, LCA, and TCA significantly increased in the model group. Compared with the model group, these bile acids showed an obvious callback in the M_EK and M_VEK groups. UDCA and LCA were presented with an upward trend, while TCA and DCA were presented with a downward trend after the intervention of UDCA. Compared with the M_EK and the M_VEK groups, respectively, there were no significant differences between the M_EK_UDCA and M_VEK_UDCA groups. These results indicate that EK and VEK could alleviate MAE by synergistically improving the disorder of the fecal composition of bile acids.

### 3.10 Targeted microbial analysis of gut microbiota in EK/VEK-treated MAE rats with UDCA intervention

**FIGURE 8:**
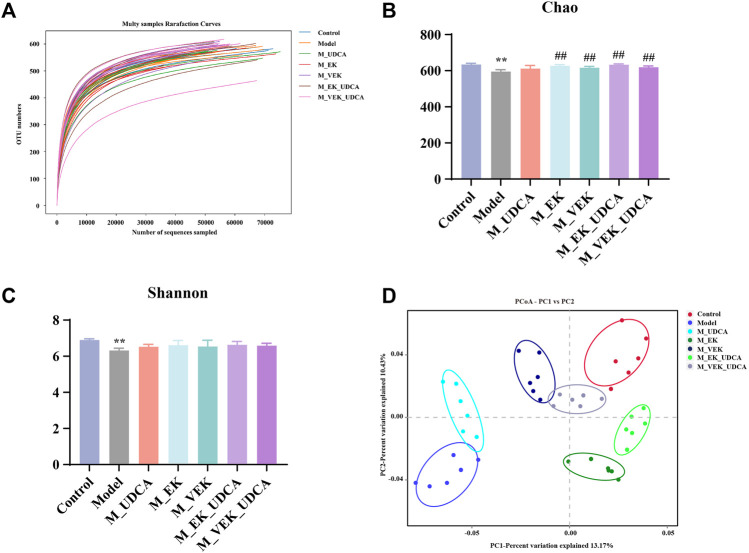
Diversity and richness of the intestinal flora of rats. **(A)** OTU rarefaction curve, **(B)** Shannon index, **(C)** Chao index, and **(D)** PCoA to evaluate beta diversity. **p <0.01, compared to the control group; ^**##**^ *p* <0.01, compared to the model group.

The composition of the gut microbiota was analyzed using the 16S rRNA gene in the model rats, and the structural changes in the gut microbiota were investigated when the rats were treated with different doses. OTU rarefaction analysis showed that although several new OTUs could be obtained by additional sequencing, the amount of sequencing data was reasonable and sufficient (Figure 8A). Compared to the control group, MAE rats showed decreased microbial diversity and richness concerning the Chao and Shannon indexes. Significant increases in the Chao index and the increasing trend in the Shannon index, indicating a higher microbiota community diversity, were observed after the treatments with EK/VEK (Figures 8B, C). The UDCA intervention showed no influence on bacterial diversity. Beta diversity and the overall structure of the gut microbiota were then analyzed using UniFrac distance-based principal coordinate analysis (PCoA) (Figure 8D). The model group was located far away from the control group. This indicated that the composition of the gut microbiota in MAE rats differed from the control group. The M_UDCA group was close to the model group. M_EK, M_VEK, M_EK_UDCA, and M_VEK_UDCA groups located between the control group and the model group. Therefore, the M_EK_UDCA and M_VEK_UDCA groups were close to the M_EK group and M_VEK groups, respectively. EK and VEK, with the intervention of UDCA, induced obvious changes in the gut microbiota composition of the MAE rats, which were much closer to the control group.

**FIGURE 9:**
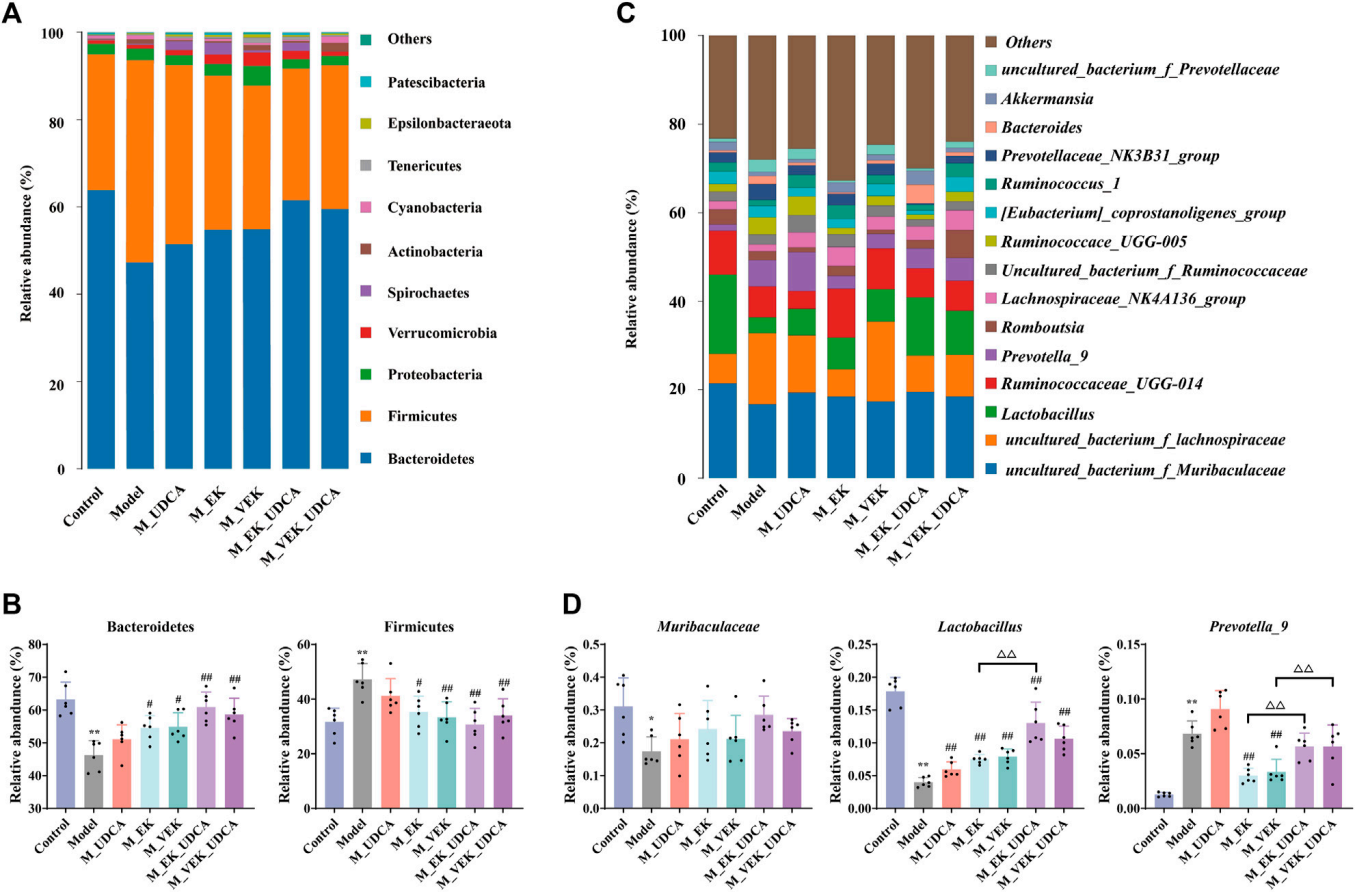
Relative abundance of the microbial species in different groups. **(A, B)** Phylum-level analysis of Bacteroidetes and Firmicutes; **(C, D)** genus-level analysis of Muribaculaceae, *Lactobacillus*, and *Prevotella_9* (top three differential taxa). *p <0.05, **p <0.01, compared to the control group; ^**#**^ *p* <0.05, ^**##**^ *p* <0.01, compared to the model group; ^Δ^ *P* <0.05, ^ΔΔ^ *p* <0.01, compared to EK; and ^▽^ *p* <0.05, compared to the VEK group.

An overview of the gut microbiota composition at the phylum level is presented in Figures 9A, B. Compared to the control group, the abundance of Firmicutes increased and that of Bacteroidetes decreased in the model group. EK and VEK significantly reduced the abundance of Firmicutes and increased Bacteroidetes in the MAE rats. The UDCA intervention was presented with a synergistic trend with EK/VEK. In Figures 9C, D, the OTUs assigned to Muribaculaceae were decreased in the MAE rats at the family level. It was increased in all the administration groups. Regarding the genus level, the abundance of *Lactobacillus* decreased and that of *Prevotella_9* increased in the model rats compared to the control group. *Lactobacillus* was presented with an enrichment trend under the treatments of EK and VEK, which was further enriched by UDCA intervention. *Prevotella_9* was decreased under the treatments of EK/VEK but was enriched by the administration of UDCA. These results suggest that MAE seriously disrupted the balance of the gut microbiota in the rats. The intervention of UDCA synergistically improved the rebalance of intestinal bacterial homeostasis induced by MAE in the treatment of EK/VEK.

## 4 Discussion

Previous studies indicated that the water-expelling effect of VEK was dosage-independent. A favorable pharmacological effect was observed when malignant ascites rats were orally administered at a dose of 680 mg·kg^−1^ (eight times the clinical dosage, Guo et al., 2021). After EK was stir-fried with vinegar, the intestinal toxicity of VEK in normal rats was significantly decreased, but with retained water-expelling activity against malignant ascites effusion (Zhang et al., 2016; Jiang et al., 2018). The water-expelling effect of EK and VEK was closely related to the gut microbiota and its co-metabolism with the host (Guo et al., 2021). BAs in organisms are synthesized in the liver and are mainly metabolized in the intestine. Abnormal changes in their composition and content are usually associated with liver- and intestine-related diseases (Zhang, S. et al., 2021). For example, the severity of colitis in mice was positively correlated with fecal bile acid hydrophobicity and DCA concentration (Stenman et al., 2013). The fewer hydroxyl groups in the bile acid structure, the higher the hydrophobicity and the higher the apoptotic rate of colonic epithelial cells (Staley et al., 2017). UDCA was recognized for its anti-inflammatory and cytoprotective effects, in addition to liver and intestinal protection (Van den Bossche et al., 2017; Ward et al., 2017). The increase in intestinal permeability was mainly related to changes in the gut microbiota, and intestinal permeability may be influenced by bile acid metabolism. At the same time, the composition of bile acids was regulated by the gut microbiota. The increase in the size of the bile acid pool may also inhibit the abundance of beneficial bacteria in the intestine, causing an imbalance and affecting the diversity of the gut microbiota (Stenman et al., 2012).

Therefore, a sensitive and rapid UPLC-MS/MS method was developed for the quantitative detection of 15 bile acids in feces. The contents in the rat feces of different groups were determined. MAE caused a significant increase in the contents of DCA, LCA, and TCA, which were reduced after the administration of EK/VEK. The bacterial metabolism of abnormally high concentrations of secondary bile acids, mainly DCA and LCA, can trigger excessively harmful effects on the intestinal mucosa, such as oxidative stress and inflammation, which exert harmful effects on the structure and function of the intestinal epithelium (Kim et al., 2018). TCA has the potential to stimulate enterobacteria that could convert taurine and cholic acid into hydrogen sulfide and deoxycholic acid, respectively, exhibiting toxic effects and promoting tumor development (Ridlon et al., 2016). In addition, MAE caused a significant decrease in UDCA, which was an endogenous hydrophilic secondary bile acid that could stimulate bile flow and protect liver cells from membrane-destructive bile acids (such as LCA) (Zhang et al., 2019). UDCA may alleviate the barrier dysfunction caused by DCA and inhibit DCA-induced cell apoptosis (Staley et al., 2017). The reduction in UDCA suggests that the composition of the fecal bile acid is developing in an unhealthy direction.

On this basis, to further confirm the correlation between intestinal flora and host co-metabolites, UDCA was selected to intervene in MAE rats to investigate whether the water-expelling effect of EK/VEK was mediated by bile acids. Compared to the control group, after the establishment of MAE, ascites were generated, accompanied by damage to the liver, stomach, and intestine. The expression of intestinal AQP3 protein was also significantly upregulated in MAE rats. AQP3 is considered an important potential indicator related to the inflammatory response, and the promotion of AQP3 protein expression inhibition may induce the release of IL-6 and TNF-*α* (da Silva et al., 2021). EK and VEK could promote urine excretion, reduce the number of ascites, increase the water content of feces, and reduce liver, stomach, and intestinal damage in MAE rats. MAE rats treated with UDCA were similar to those in the model group. When EK/VEK was administered to MAE rats with UDCA intervention, the aforementioned indicators of the rats improved significantly and were even slightly better than those administered with EK/VEK alone, suggesting that EK/VEK and bile acids had a synergistic water-expelling effect on MAE rats. The analysis of fecal bile acid composition showed that UDCA intervention could enhance the decreased content of UDCA and increased content of DCA and TCA in MAE rats administered with EK/VEK. The increase in hydrophilic bile acid UDCA could alleviate the liver and gastrointestinal damage caused by ascites by diluting hydrophobic bile acids in the bile acid pool (Kim et al., 2018). However, UDCA intervention further increased LCA, which may be due to the slow and incomplete absorption of UDCA (Sinakos et al., 2010; Ward et al., 2017).

The analysis of the gut microbiota composition showed that the overall structure of the gut microbiota of MAE rats significantly varied from that of normal rats, and EK/VEK restored the intestinal microbial composition to a pattern similar to that of normal rats. The abundance of *Lactobacillus* and Muribaculaceae decreased significantly, and the abundance of *Prevotella_9* increased significantly in the MAE rats. EK/VEK could improve the disrupted gut microbiota to varying degrees. UDCA intervention did not show any influence on the number of species and diversity of the gut microbiota but showed a synergistic callback in the disordered abundance of bacteria, such as *Lactobacillus* and Muribaculaceae. *Lactobacillus* belongs to the Firmicutes and is a representative beneficial bacterium for maintaining gut homeostasis (Yadav and Chauhan, 2022). A lower abundance of *Lactobacillus* was observed in MAE rats, which was in accordance with the previous studies (Rodríguez-Nogales et al., 2017; Guo et al., 2021). *Lactobacillus* has an important bile salt hydrolase (BSH) activity, and its lower abundance could cause a decrease in the secretion of BSH, leading to a significant increase in fecal TCA (Winston and Theriot, 2020). Muribaculaceae (belonging to Bacteroidetes) was positively correlated with the function of the inner mucus layer, which is a critical barrier for protecting the intestinal epithelium barrier (Volk et al., 2019). The abundance of *uncultured_bacterium_f_Lachnospiraceae* in each administration group also showed an upward trend. The abundance of Muribaculaceae was negatively correlated with DCA (Staley et al., 2017), and certain bacteria belonging to the Lachnospiraceae may metabolize primary bile acids to secondary bile acids (MengYuan and XiaoXing, 2020). Compared with the rats in the non-intervention groups, the abundance of *Prevotella_9*, which used mucin as a growth substrate in the UDCA intervention groups, increased (Derrien et al., 2010; Van den Bossche et al., 2017). This indicates that the administration of UDCA protected the gastrointestinal tract from stimulation. These results suggest that UDCA played a synergistic role along with EK and VEK in modulating the gut microbiota toward a normal state.

## 5 Conclusion

In summary, EK and VEK exerted water-expelling effects in MAE rats, protecting the liver and intestine. No significant differences were found between EK and VEK. The water-expelling effect of VEK did not directly depend on the BA-mediated gut microbiota. However, EK/VEK and BAs had synergistic effects on malignant ascites effusion by regulating the gut microbiota. These results provided a scientific basis for the water-expelling effect of VEK and the novel treatment strategy of combining VEK and UDCA.

## Data Availability

The original contributions presented in the study are included in the article/Supplementary Material, further inquiries can be directed to the corresponding authors.
