# Supplementary material for: Synergistic effect of Euphorbia kansui stir-fried with vinegar and bile acids on malignant ascites effusion through modulation of gut microbiota
Source: Front Pharmacol. 2023 Nov 9;14:1249910. doi: 10.3389/fphar.2023.1249910 (PMC10665909; doi:10.3389/fphar.2023.1249910)
Supplement: Supplementary file 1 [file Table1.doc]

Supplementary Material

# Synergistic Effect of *Euphorbia Kansui* Stir-fried with Vinegar and Bile Acids on Malignant Ascites Effusion through Modulating Gut Microbiota

Shengyun Dai ^1†^, Shikang Zhou ^2†^, Yonghui Ju ^3^, Weifeng Yao ^2^, Yuping Tang ^4^, Jian Zheng ^1^, Shuangcheng Ma ^1^, Yi Zhang ^2*^, Li Zhang ^2*^

*** Correspondence:** Yi Zhang: zhangyi@njucm.edu.cn, Li Zhang: zhangli@njucm.edu.cn

**Table S1 Inter-day and intra-day precision**

|  | Theoretical concentration (ng•mg^-1^) | Inter-day  RSD (%) | Intra-day  RSD (%) |
| --- | --- | --- | --- |
| *α*-MCA | 41.71 | 7.22 | 6.17 |
|  | 83.67 | 5.49 | 6.90 |
|  | 125.61 | 7.47 | 9.61 |
| *β*-MCA | 32.76 | 14.50 | 8.89 |
|  | 65.74 | 10.25 | 10.77 |
|  | 98.68 | 6.37 | 7.94 |
| *ω*-MCA | 26.20 | 8.39 | 7.95 |
|  | 52.55 | 4.22 | 9.11 |
|  | 78.90 | 7.40 | 10.72 |
| UDCA | 19.31 | 12.58 | 7.35 |
|  | 38.74 | 5.64 | 8.24 |
|  | 58.16 | 8.37 | 7.28 |
| HDCA | 184.42 | 8.23 | 4.12 |
|  | 369.99 | 7.25 | 7.63 |
|  | 555.46 | 10.40 | 7.08 |
| CA | 5.93 | 11.26 | 5.73 |
|  | 11.63 | 4.14 | 10.15 |
|  | 17.18 | 8.99 | 6.11 |
| LCA | 52.91 | 7.47 | 12.66 |
|  | 106.18 | 10.16 | 9.40 |
|  | 159.41 | 4.26 | 9.78 |
| DCA | 121.48 | 7.47 | 9.62 |
|  | 243.73 | 5.17 | 9.44 |
|  | 365.90 | 6.23 | 8.79 |
| CDCA | 0.38 | 8.97 | 8.96 |
|  | 0.76 | 10.28 | 10.18 |
|  | 1.13 | 6.91 | 10.51 |
| T-*β*-MCA | 1.00 | 9.24 | 4.25 |
|  | 2.02 | 5.28 | 7.90 |
|  | 3.03 | 3.67 | 7.84 |
| T-*α*-MCA | 0.20 | 4.62 | 4.53 |
|  | 0.41 | 3.20 | 6.65 |
|  | 0.61 | 6.67 | 6.77 |
| TCA | 1.05 | 9.62 | 9.31 |
|  | 2.11 | 2.72 | 10.02 |
|  | 3.17 | 9.88 | 4.20 |
| GCA | 0.25 | 13.65 | 6.28 |
|  | 0.50 | 6.87 | 9.47 |
|  | 0.75 | 4.92 | 8.28 |
| GDCA | 0.09 | 9.48 | 10.87 |
|  | 0.19 | 6.79 | 6.60 |
|  | 0.28 | 5.98 | 7.66 |
| TUDCA | 0.08 | 8.32 | 10.56 |
|  | 0.16 | 12.25 | 7.63 |
|  | 0.24 | 7.42 | 6.69 |

**Table S2 Stability investigation (*n* = 3)**

|  | Theoretical concentration (ng•mg^-1^) | Accuracy (%) | | | | | RSD (%) |
| --- | --- | --- | --- | --- | --- | --- | --- |
|  |  | 0 h | 24 h | -80 ℃ freeze-thaw 3 times | | -80 °C  storage  15 d |  |
| *α*-MCA | 41.71 | 105.72 | 92.08 | 85.98 | 89.09 | | 9.33 |
|  | 83.67 | 106.68 | 96.48 | 95.88 | 84.96 | | 9.24 |
|  | 125.61 | 89.05 | 88.69 | 91.50 | 85.65 | | 2.70 |
| *β*-MCA | 32.76 | 94.52 | 86.17 | 85.04 | 82.05 | | 6.14 |
|  | 65.74 | 90.57 | 97.57 | 99.34 | 90.16 | | 5.60 |
|  | 98.68 | 94.30 | 86.57 | 84.60 | 80.94 | | 6.51 |
| *ω*-MCA | 26.20 | 93.11 | 86.13 | 83.23 | 86.59 | | 4.78 |
|  | 52.55 | 80.87 | 82.77 | 82.59 | 86.17 | | 2.67 |
|  | 78.90 | 91.87 | 92.84 | 82.11 | 89.05 | | 5.44 |
| UDCA | 19.31 | 103.82 | 86.84 | 89.39 | 88.60 | | 8.51 |
|  | 38.74 | 108.28 | 95.30 | 91.55 | 91.27 | | 8.28 |
|  | 58.16 | 99.27 | 94.47 | 92.31 | 96.08 | | 3.07 |
| HDCA | 184.42 | 105.60 | 89.74 | 87.14 | 89.04 | | 9.21 |
|  | 369.99 | 102.92 | 88.48 | 84.65 | 89.62 | | 8.71 |
|  | 555.46 | 112.00 | 107.24 | 101.94 | 91.23 | | 8.65 |
| CA | 5.93 | 108.13 | 105.49 | 109.06 | 80.89 | | 2.58 |
|  | 11.63 | 112.24 | 103.50 | 99.47 | 80.85 | | 5.07 |
|  | 17.18 | 115.46 | 105.89 | 104.14 | 114.92 | | 5.38 |
| LCA | 52.91 | 109.63 | 107.81 | 93.41 | 105.86 | | 7.05 |
|  | 106.18 | 105.56 | 106.45 | 89.86 | 86.92 | | 10.54 |
|  | 159.41 | 98.86 | 94.28 | 87.40 | 86.00 | | 6.57 |
| DCA | 121.48 | 100.78 | 94.54 | 98.16 | 92.88 | | 3.15 |
|  | 243.73 | 96.52 | 95.84 | 93.36 | 96.57 | | 1.58 |
|  | 365.90 | 96.23 | 87.70 | 92.10 | 83.24 | | 6.24 |
| CDCA | 0.38 | 96.29 | 100.44 | 85.59 | 89.99 | | 7.08 |
|  | 0.76 | 86.55 | 86.30 | 84.88 | 84.55 | | 1.17 |
|  | 1.13 | 92.99 | 92.66 | 85.32 | 89.66 | | 3.95 |
| T-*β*-MCA | 1.00 | 99.99 | 93.51 | 85.15 | 81.68 | | 9.17 |
|  | 2.02 | 92.94 | 86.93 | 88.33 | 85.16 | | 3.77 |
|  | 3.03 | 93.78 | 84.21 | 87.74 | 85.94 | | 4.74 |
| T-*α*-MCA | 0.20 | 91.36 | 94.73 | 86.87 | 89.51 | | 3.65 |
|  | 0.41 | 87.67 | 88.65 | 89.33 | 81.68 | | 4.03 |
|  | 0.61 | 92.01 | 89.54 | 93.22 | 84.52 | | 5.78 |
| TCA | 1.02 | 87.24 | 85.04 | 90.95 | 96.40 | | 5.52 |
|  | 2.03 | 93.02 | 89.32 | 94.91 | 87.39 | | 3.76 |
|  | 3.05 | 96.22 | 96.07 | 85.65 | 87.30 | | 6.16 |
| GCA | 0.25 | 102.18 | 93.16 | 99.69 | 90.17 | | 5.80 |
|  | 0.50 | 92.67 | 96.81 | 98.66 | 94.52 | | 2.74 |
|  | 0.75 | 105.74 | 97.65 | 87.81 | 89.28 | | 8.72 |
| GDCA | 0.09 | 101.11 | 82.58 | 92.98 | 93.18 | | 8.22 |
|  | 0.19 | 96.70 | 85.66 | 84.51 | 80.48 | | 7.99 |
|  | 0.28 | 83.92 | 80.87 | 85.81 | 80.79 | | 2.96 |
| TUDCA | 0.08 | 90.89 | 96.79 | 93.46 | 90.46 | | 3.13 |
|  | 0.16 | 93.43 | 86.18 | 87.60 | 87.43 | | 3.66 |
|  | 0.24 | 87.04 | 89.08 | 86.17 | 81.96 | | 3.48 |

**Table S3 Absolute recovery of bile acids in feces (*n* = 5)**

|  | Theoretical concentration (ng•mg^-1^) | Measured concentration  (ng•mg^-1^) | Recovery (%) | RSD (%) |
| --- | --- | --- | --- | --- |
| *α*-MCA | 41.71 | 47.81 | 114.6 | 7.75 |
|  | 83.67 | 85.80 | 102.6 | 8.84 |
|  | 125.61 | 114.75 | 91.3 | 13.70 |
| *β*-MCA | 32.76 | 30.49 | 91.9 | 9.12 |
|  | 65.74 | 61.75 | 93.9 | 2.73 |
|  | 98.68 | 82.11 | 83.2 | 9.77 |
| *ω*-MCA | 26.20 | 22.79 | 87.0 | 7.70 |
|  | 52.55 | 44.76 | 85.2 | 6.90 |
|  | 78.90 | 69.65 | 88.3 | 11.09 |
| UDCA | 19.31 | 15.98 | 82.8 | 9.77 |
|  | 38.74 | 35.62 | 91.9 | 3.02 |
|  | 58.16 | 53.91 | 92.7 | 12.25 |
| HDCA | 184.42 | 165.04 | 89.5 | 7.52 |
|  | 369.99 | 366.41 | 99.0 | 6.16 |
|  | 555.46 | 563.69 | 101.5 | 1.80 |
| CA | 5.93 | 6.77 | 114.1 | 4.81 |
|  | 11.63 | 12.73 | 109.5 | 1.83 |
|  | 17.18 | 18.20 | 105.8 | 4.20 |
| LCA | 52.91 | 54.08 | 102.3 | 13.28 |
|  | 106.18 | 111.16 | 104.7 | 6.49 |
|  | 159.41 | 152.49 | 95.7 | 11.28 |
| DCA | 121.48 | 115.12 | 94.8 | 5.01 |
|  | 243.73 | 231.76 | 95.1 | 3.77 |
|  | 365.90 | 335.87 | 91.8 | 6.41 |
| CDCA | 0.38 | 0.34 | 89.20 | 5.72 |
|  | 0.76 | 0.64 | 84.31 | 2.82 |
|  | 1.13 | 1.02 | 90.04 | 6.61 |
| T-*β*-MCA | 1.00 | 0.90 | 89.8 | 7.62 |
|  | 2.02 | 1.71 | 84.9 | 9.82 |
|  | 3.03 | 2.56 | 84.5 | 11.83 |
| T-*α*-MCA | 0.20 | 0.18 | 86.0 | 5.07 |
|  | 0.41 | 0.36 | 88.6 | 2.51 |
|  | 0.61 | 0.56 | 91.8 | 12.01 |
| TCA | 1.02 | 0.85 | 80.7 | 7.63 |
|  | 2.03 | 2.08 | 98.9 | 4.96 |
|  | 3.05 | 2.84 | 89.8 | 5.04 |
| GCA | 0.25 | 0.22 | 89.1 | 7.56 |
|  | 0.50 | 0.46 | 92.4 | 3.53 |
|  | 0.75 | 0.65 | 85.6 | 11.82 |
| GDCA | 0.09 | 0.09 | 96.8 | 12.65 |
|  | 0.19 | 0.16 | 85.2 | 9.21 |
|  | 0.28 | 0.25 | 86.4 | 8.81 |
| TUDCA | 0.08 | 0.08 | 102.5 | 3.35 |
|  | 0.16 | 0.14 | 89.5 | 6.65 |
|  | 0.24 | 0.20 | 83.9 | 4.55 |
